# The Radical Cure of Hernia

**Published:** 1893-11-25

**Authors:** 


### THE RADICAL CURE OF HERNIA.

Under what circumstances should we recommend the operation of radical cure to patients with hernia? Many persons with rupture may desire to be operated on and cured, in whom a well-fitting truss will easily keep up the rupture. In such cases we should hardly be justified in performing a radical cure, for although with strict antiseptic precautions the risk is almost nil, yet these precautions may fail occasionally, and thus allow a minimum of risk to creep in. And after all many people get so accustomed to a truss, when it fits well, that it is no burden to them to wear one. However, if the patient understands that the operation is not quite free from risk, and is prepared to run that risk rather than have the slight trouble of wearing a truss, the question of operation is rather one for his decision than ours. But what then are the cases really suitable for radical cure. 1st. Cases where the hernia cannot be kept up by a well-fitting truss. Perhaps the truss answers well enough under all ordinary circumstances, but with any increased exertion allows the hernia to descend. 2nd. Cases of hernia in sailors and others who are at times in places where they cannot get a truss mended or procure another, and yet where they are engaged in very violent labour at times. 3rd. Cases of hernia (chiefly femoral), where a small piece of omentum is irreducible, and will not allow a truss to press firmly and evenly on the ring, so that bowel may come down and become strangulated. 4th. Irreducible hernias which are a burden to the patient, and into which a little more bowel may descend and become strangulated.

But we must remember that some of these very large irreducible hernias are really dangerous cases for operation. The adhesions in them are often most extensive, and the operations frequently very prolonged and difficult.

Some surgeons operate on the ruptures of children. In the case of children of the poor, in instances where it is evident that the likelihood of their wearing a truss constantly is very little, perhaps we shall do the best for them by performing a radical cure. But we must not forget that in early childhood a truss constantly worn not only keeps back the hernia, but by pressure on the inguinal canal causes adhesion, and thus brings about a radical cure itself.

And now we come to a very important question with regard to radical cure of hernia. Is the cure really a radical and permanent one? The answer to this will depend, we believe, very much on the method of operating. Macewen's method has been in his hands most thoroughly radical, the patients going about their work without the need of any truss. Some other methods have been less so; in fact, the surgeons who have introduced them have said that the cure obtained thereby was not really a "radical" cure at all, and they have recommended the continued use of a truss after operating, though it was then worn with the efficiency before unattainable. Other surgeons consider that the wearing of a truss after radical cure not only ought not to be necessary but is actually harmful, the pressure of the truss tending rather to cause absorption of the exudation which has closed the canal. Possibly, in some cases, although a truss need not be worn, and may even do harm, yet a light support may be employed with advantage when very violent labour is engaged in.

The older methods of radical cure are now practically abandoned in favour of the modern. The days of Wood's subcutaneous wiring of the pillars of the ring, and similar operative procedures, are over. With the fear of opening the peritoneum removed by the use of antiseptics in surgery, a method which allows the surgeon to see what he is doing (or, at any rate, to feel), has replaced the less satisfactory plan to working subcutaneously.

Of all the modern, or "open" methods as they are called, Macewen's is the most thorough. He dissects the sac out of the scrotum, and uses this as a plug on the peritoneal aspect of the internal ring, and then brings the sides of the inguinal canal together by deep sutures. His operation is only applicable to inguinal hernia. Banks has been content to ligature the sac at the neck, cut away the rest of the sac, and then stitch up the ring. But there are various modifications of the operation, known by the names of the surgeons who have practised them. The principal of all these open operations is obliteration of the sac and the internal ring. Keatly has tried in several cases injections into the sac of irritating fluids, so as to cause inflammatory exudation about the neck, and thus its obliteration; but his method is uncertain, and certainly not quite free from danger.

In femoral hernia it is less easy to perform a radical cure than in inguinal. Most surgeons have been content with ligature of the neck of the sac, or twisting the sac according to Ball's method, and have made no attempt to bring the edges of the ring together; indeed, from the anatomical nature of the ring it would be impossible to approximate its edges. It has, however, lately been proposed to unite the structures on the distal side of the ring so as to close up the canal, but the tension on them when united would be so great as to make it very doubtful whether the union would hold. However, the simpler proceeding of ligaturing the neck of the sac has given satisfactory results in many cases. It has usually been done after operation for strangulated hernia. Every strangulated hernia operation should, if the condition of the bowel is satisfactory and the state of the patient admits of it, be followed by radical cure of the hernia.

